# Angiogenesis Inhibitor Vasohibin-1 Enhances Stress Resistance of Endothelial Cells via Induction of SOD2 and SIRT1

**DOI:** 10.1371/journal.pone.0046459

**Published:** 2012-10-08

**Authors:** Hiroki Miyashita, Tatsuaki Watanabe, Hideki Hayashi, Yasuhiro Suzuki, Takanobu Nakamura, Soichi Ito, Manabu Ono, Yasushi Hoshikawa, Yoshinori Okada, Takashi Kondo, Yasufumi Sato

**Affiliations:** 1 Department of Vascular Biology, Institute of Development, Aging and Cancer, Tohoku University, Sendai, Japan; 2 Department of Thoracic Surgery, Institute of Development, Aging and Cancer, Tohoku University, Sendai, Japan; The University of New South Wales, Australia

## Abstract

Vasohibin-1 (VASH1) is isolated as an endothelial cell (EC)-produced angiogenesis inhibitor. We questioned whether VASH1 plays any role besides angiogenesis inhibition, knocked-down or overexpressed VASH1 in ECs, and examined the changes of EC property. Knock-down of VASH1 induced premature senescence of ECs, and those ECs were easily killed by cellular stresses. In contrast, overexpression of VASH1 made ECs resistant to premature senescence and cell death caused by cellular stresses. The synthesis of VASH1 was regulated by HuR-mediated post-transcriptional regulation. We sought to define the underlying mechanism. VASH1 increased the expression of (superoxide dismutase 2) SOD2, an enzyme known to quench reactive oxygen species (ROS). Simultaneously, VASH1 augmented the synthesis of sirtuin 1 (SIRT1), an anti-aging protein, which improved stress tolerance. Paraquat generates ROS and causes organ damage when administered *in vivo*. More *VASH1 (+/−)* mice died due to acute lung injury caused by paraquat. Intratracheal administration of an adenovirus vector encoding human VASH1 augmented SOD2 and SIRT1 expression in the lungs and prevented acute lung injury caused by paraquat. Thus, VASH1 is a critical factor that improves the stress tolerance of ECs via the induction of SOD2 and SIRT1.

## Introduction

Endothelial cells (ECs) are multifunctional cells covering the entire luminal surface of all blood vessels. They form an interface between the circulating blood in the lumen and the rest of the vessel wall, and maintain vascular homeostasis. ECs control the transport of various molecules across the vascular wall, regulate immune response via the adhesion of leukocytes to the vessel wall for extravasation, manipulate vascular tonus, and prevent thrombotic events. When stimulated by angiogenic factors, ECs form neo-vessels. During the course of this process, termed angiogenesis, ECs produce molecules that control angiogenesis in an auto-regulatory manner. Endothelial tip cells produce delta-like 4, which controls the number of subsequent tips via binding to Notch1 on stalk cells [1]. We recently identified vasohibin-1 (VASH1) as an inhibitor of angiogenesis. VASH1 is expressed in ECs, whose expression is enhanced during angiogenesis, and that terminates angiogenesis as an autocrine manner [2], [3].

The vascular system is one of the main target organs of aging. Age-related vascular diseases are the consequence of endothelial damage, and one of the major causes of this damage is oxidative stress [4]. When subjected to oxidative stress, cells generally exit the cell cycle and undergo premature senescence. Replicative senescence is associated with the shortening of telomeres and reduced telomerase activity, whereas premature senescence does not require those events. The oxidative stress-induced premature senescence of ECs is thought to play important roles in the pathogenesis of age-related vascular diseases, as premature senescence of ECs occurs in the vasculature of individuals who are more susceptible to develop atherosclerosis [5], [6].

With respect to angiogenesis regulators, angiogenesis inhibitors generally induce EC death and vascular regression. It was recently described that one of the detectable indicators of dysfunctional senescent ECs is collagen XVIII and its C-terminal anti-angiogenic fragment, known as endostatin. Moreover, an increase in the level of endostatin exacerbates vascular damage, thus triggering a vicious cycle [7].

Here we examined the function of VASH1. As VASH1 also has anti-angiogenic activity, it may affect vascular damage. However, to our surprise, VASH1 actually enhanced the maintenance of ECs by strengthening their resistance to oxidative or serum-starvation-induced stress. The significance of this effect and the underlying mechanism is examined in this study.

## Materials and Methods

All of the animal studies were reviewed and approved by the Center for Laboratory Animal Research, Tohoku University in accordance with established standards of humane handling of research animals.

### Materials

The following materials and their sources were used: α-minimal essential medium (αMEM) and Dulbecco-modified Eagle medium (DMEM) from Wako Pure Chemical Industries, Ltd. (Osaka, Japan); Superscript One-step RT-PCR with platinum Taq, Lipofectamine RNAi max, Opti-MEM I, stealth siRNAs, and 5–6-chloromethyl-2′, 7′-dichlorodihydro-fluorescein diacetate, acetyl ester (CM-H2DCFDA) from Invitrogen (Carlsbad, CA); endothelial basal medium (EBM) and endothelial cell growth supplements from Clonetics (Walkersville, MD); Isogen from Nippon Gene (Toyama, Japan); Hybond-ECL from Amersham (Buckinghamshire, UK); N-acetylcysteine (NAC), SU5416, vascular endothelial growth factor (VEGF), protein G Sepharose, anti- β-actin antibody from Sigma (St. Louis, Mo); hydrogen peroxide from Mitsubishi Chemical Corporation (Tokyo, Japan); anti-8-hydroxydeoxyguanosine (8-OHdG) antibody from Abcam (Cambridge, MA); anti-silent mating type information regulation 2 homolog 1 (SIRT1) antibody, anti-super oxide dismutase 2 (SOD2) antibody, anti-HuR antibody, ataxia teleangiectasia mutation (ATM) antibody, phospho-ATM antibody (Ser1981), anti-rabbit IgG and SIRT1 activator 3 from Santa Cruz Biotechnology (Santa Cruz, CA); and anti-light chain 3 (LC3) antibody from Medical & Biological Laboratory (Nagoya, Japan). Horseradish peroxidase (HRP)-conjugated anti-human VASH1 mAb (4E12) was described previously [2].

### Cells

Human umbilical vein endothelial cells (HUVECs) and human aortic endothelial cells (HAECs) were obtained from Sanko Junyaku Industries (Tokyo, Japan) and were cultured on type I collagen-coated dishes (Iwaki, Chiba, Japan) in EBM containing endothelial cell growth supplements and 2% fetal bovine serum (FBS). All experiments using HUVECs and HAECs were performed at population doubling levels of less than 10. Normal human bronchial epithelial cells (NHBECs) were obtained from Lonza (Basel, Switzerland) and were cultured in BEGM Bullet Kit (Lonza). Mouse EC line MS1, a cell line immortalized from pancreatic ECs by SV40 large T antigen, were purchased from American Type Culture Collection (ATCC, Manassas, VA). The MS1 cells were cultured in αMEM supplemented with 10% FBS, as described previously [8].

### VASH1 overexpression in HUVEC and MS1

VASH1 overexpression in human unblical vein endothelial cells (HUVECs) or in human aortic endothelial cells (HAECs) was achieved by infection with a non-proliferative adenovirus vector encoding human VASH1 (AdVASH1) at a final multiplicity of infection of 30 [2]. Alternatively, HUVECs were transiently transfected with the VASH1 expression plasmid vector [8]. VASH1 overexpressing MS1 stable clones were described previously [8].

### Reverse transcriptase-polymerase chain reaction (RT-PCR)

Total RNAs were extracted and RT-PCR was performed by using a One-step RT-PCR kit (Invitrogen) according to manufacturer's instructions. Primer pairs used in this study were as follows; human VASH1 forward, 5′- ATG GAC CTG GCC AAG GAA AT-3′,and reverse, 5′- CAT CCT TCT TCC GGT CCT TG-3′;human NOX1 forward, 5′-CGT CTG CTC TCT GCT TGA AT-3′, and reverse, 5′-TGA ATC CCT AAG CCA AGG AT-3′; human NOX2 forward, 5′-GTC TGG TAT TAC CGG GTT TA-3′, and reverse, 5′-GTG CTA CTG AAT AAG GAT CAG-3′; human NOX4 forward, 5′-ATG GCT GTG TCC TGG AGG AG-3′, reverse, 5′-GAT CAT GAG GAA TAG CAC CA-3′; human SOD1 forward, 5′-AAG GAC TGA CTG AAG GCC TG-3′, and reverse, 5′-AAG CCA AAC GAC TTC CAG CG-3′; human SOD2 forward, 5′-CAG GCA GCT GGC TCC GGT TT-3′, and reverse, 5′-TGC AGT GGA TCC TGA TTT GG-3′; human SOD3 forward, 5′-ATG CTG GCG CTA CTG TGT TC-3′, and reverse, 5′-TTC CCG TTC TCC ACG CTG GC-3′; human catalase forward, 5′-ACC AGA TGC AGC ACT GGA AG-3′, and reverse, 5′-GGG GGT GTT ATT TCC AA CGA-3′; human G3PDH forward, 5′-ACC ACA GTC CAT GCC ATC AC-3′and reverse 5′- TCC ACC ACC CTG TTG CTG TA-3′.

### Senescence associated β-galactosidase (SA β-gal) staining

SA beta-gal was determined by using a senescence detection kit (Abcam) according to the manufacturer's instructions. Briefly, cells were incubated overnight in freshly prepared staining solution (containing 1 µg/ml X-gal) at 37°C. The percentage of senescent cells was obtained by counting the number of blue-stained cells and the total cells per field under an inverted microscope.

### Gene silencing by stealth siRNA

HUVECs or HAECs were transfected with synthetic siRNAs in Lipofectamine RNAi max containing Opti-MEM I at a final concentration of 10 nmol/L. At 12 hour post-transfection, the cell culture medium was replaced with growth medium; and the cells were then incubated for and additional 12 hours prior to use in experiments. Specific gene silencing was verified by RT-PCR and Western blot analysis. The nucleotide sequences of stealth siRNAs used in this study are as follow: for human VASH1 and its control, 5′-CAA GGA CCG GAA GAA GGA UGU UUC U-3′ and 5′-CAA CCA AGG AGA GGA GUA UUG GUC U-3′; for human HuR and its control, 5′-CGG GAU AAA GUA GCA GGA CAC AGC U -3′ and 5′-CGG AAA UGA UGG GAC CAC AAA GGC U -3′; for human SIRT1 and its control, 5′-CAG GUU GCG GGA AUC CAA AGG AUA A-3′ and 5′-CAG GCG UAA GGA CCU GGA AAU GUA A-3′; and for human SOD2 and its control, 5′-GAG GAG AAC TCG CTT CGT ATT TGT A-3 and its control, 5′-TAC TCA AAT ACG AAG CGA GTT CCU C-3′.

### Immunocytochemical analysis

HUVECs on culture slides were transfected with VASH1 siRNA or control siRNA. At the desire times thereafter the cells were fixed with 4% paraformaldehyde at room temperature, and then rendered permeable with 0.1% NP-40 in PBS. Nonspecific binding sites were blocked with 1% BSA in PBS. Primary antibody reactions were performed overnight at 4°C with anti-LC-3 antibody at a dilution of 1∶100. Secondary antibody reactions were performed for 1 hour at room temperature with Alexa 488-conjugated goat IgG against mouse antibody (Molecular Probes, Eugene, OR) at a 1∶100 dilution with 1 µmol/L To-Pro-3 iodine (Invitrogen). The cells were observed with a Fluoview FV4000 confocal fluorescence microscope (Olympus, Tokyo, Japan).

### Western blot analysis

Western blot analysis was performed as described previously [9]. Briefly, after the poly-acrylamide gel electrophoresis and membrane transfer, the membranes were blocked for 1 hour at room temperature with Tris-HCl–buffered saline (TBS) containing 5% skim milk after the transfer, and then incubated for 1 hour at room temperature in TBS containing 0.05% Tween 20 (T-TBS), 2.5% skim milk, and one of the following antibodies: anti-human VASH1 mAb (4E12) diluted 1/500, anti-SIRT1 Ab diluted 1/500, anti-SOD2 Ab, diluted 1/500 diluted 1/500 or anti- α-actin antibody diluted 1/10,000. After the membranes had been washed 3 times with T-TBS, they incubated for 1 hour with horseradish peroxidase-conjugated protein G (Bio-Rad, Hercules, CA). They were then washed again 3 times with T-TBS, after which the blots were detected by an enhanced chemiluminescence method using an ECL Western blotting detection kit (Amersham). The results were visualized by using a LAS-4000 (Fuji Film).

### Determination of SIRT1 activity

SIRT1 activity was measured by using a SIRT1 Fluorimetric Drug Discovery Kit (Biomol International, Plymouth Meeting, PA) according to the manufacturer's instructions. Briefly, cell lysates were extracted from VASH1 overexpressing or knocked-down HUVECs, and their deacetylation activity toward a peptide comprising amino acids 379–382 of human p53 (Arg-His-Lys-Lys(epsilon-acetyl)) was measured by use of a SpectraMax M2e with excitation at 360 nm and emission at 460 nm (Molecular Devices, Tokyo, Japan)

### Chromatin immunoprecipitation (ChIP) assay

Total cell lysates (20 µg) derived from MS1 cells were immunoprecipitated with anti-rabbit IgG or anti-HuR antibody (5 µg) at 4°C for 3 hours. After that, 4 times-diluted Therma-Max UPA ProteinA (Magnabeat iIncorporated, Chiba, Ichihara, Japan) was added, and incubation was carried out at room temperature for 15 min. The complexes were collected according to the manufacturer's instructions. Total RNAs were extracted from the complexes, and RT-PCR analysis was performed as described above.

### Trypan blue exclusion assay

Cells were incubated for 5 min in a solution of 0.2% trypan blue in PBS. More than 100 cells were counted in each field, and the percentage of non-viable cells was calculated.

### Detection of cellular reactive oxygen species (ROS)

ROS was detected by using Oxiselect in vitro ROS/RNS assay kit (Cell Biolabs, San Diego, Ca) according to the manufacture's protocol. Briefly, the cell lysates (1 µg) from each treated cells were incubated with DCF-DiQxyQ for 30 min at room temperature. The fluorescence was measured by the use of SpectraMax M2e (Molecular Devices, Tokyo, Japan) with excitation at 480 nm and emission at 530 nm.

### Mouse model of acute lung injury

#### Paraquat treatment

Wild-type (WT) mice (Charles River Japan, Yokohama) and C57 BJ/6 background *VASH1* (+/−) KO mice [3], [10] of C57 BJ/6 background (9 to 10 weeks of age) were used in this study. Paraquat was dissolved in phosphate-buffered saline (5 mg/mL) and injected intraperitoneally at the dosage of 50 mg/kg body weight [11]. To evaluate the protective effect of VASH1, we used purified AdVASH1. Purified AdLacZ was used for the control [2], [12]. Mice were anesthetized with ketamine (0.1 mg/g) and xylazine (0.01 mg/g) administered by intraperitoneal injection and then intubated orotracheally with a 22-G angiocatheter. A total of 75 microl of AdVASH1 or AdLacZ, containing 1×10^9^ plaque-forming units, was injected into each mouse ototracheally [13]. Paraquat was injected intraperitoneally at the dosage of 50 mg/kg body weight 72 hours after administration of the adenovirus. The mice were followed periodically over 10 days, and the number of deaths was recorded. At the end of 10 days, all remaining mice were terminated.

#### β-gal staining

Mice were sacrificed 2 days after the intratracheal administration of PBS or AdLacZ. The pulmonary circulation was flushed with ice-cold 2% buffered paraformaldehyde, 0.2% glutaraldehyde, and 0.02% NP40 via the right ventricle. The lung blocks were inflation-fixed through the trachea with 2% buffered paraformaldehyde/PBS for 2 hours at room temperature. Thereafter, the lungs were incubated at room temperature in 1 mg/mL X-gal, 5 mmol/L potassium ferricyanide crystalline, 5 mmol/L potassium ferricyanide trihydrate, 2 mmol/L magnesium chloride, and 0.1% NP40 and then embedded in paraffin. Four-micrometer sections were prepared and then counterstained with nuclear fast red.

#### Histological analysis of the lungs

Mice were sacrificed 2 days after paraquat administration. The lungs were inflation-fixed through the trachea with 4% buffered paraformaldehyde/PBS overnight at 4°C, and then embedded in paraffin. Four-micrometer sections were prepared and then stained with hematoxylin and eosin (H&E).

For the staining of 8-OHdG, sections were incubated with the anti-8-OHdG antibody overnight at 4°C at a 1∶20 dilution. They were then incubated in 10% H_2_O_2_/methanol to block endogenous peroxidase activity. The secondary antibody reaction was performed with biotin-conjugated anti-mouse IgG for 40 min at room temperature. Streptavidin-biotin peroxidase complex formation was performed for 30 min at room temperature. The peroxidase products were visualized by using diaminobenzidine.

#### Determination of protein and cell counts in the broncheoalveolar lavage fluid (BALF)

Broncheoalveolar lavage was performed by intratracheal injection of PBS (0.7 ml) for 2 times. BALF was collected and centrifuged at 400 g for 5 min at 4°C. The recovered fluid was processed for determination of protein concentration (DC Protein Assay; Bio-Rad Laboratories, Inc, CA). The pelleted cells were resuspended in 1 ml PBS and counted.

### Calculations and statistical analysis

Data were expressed as means ± SDs. The statistical significance of differences between groups was evaluated by use of the unpaired ANOVA, and *P* values were calculated by performing the unpaired Student's *t* test. The significance between survival curves was analyzed by Kaplan-Meier survival analysis with log-rank testing. A value of *P*<0.05 was the criterion for significance.

## Results

### VASH1 protects ECs from premature senescence and stress-induced cell death

**Figure 1:**
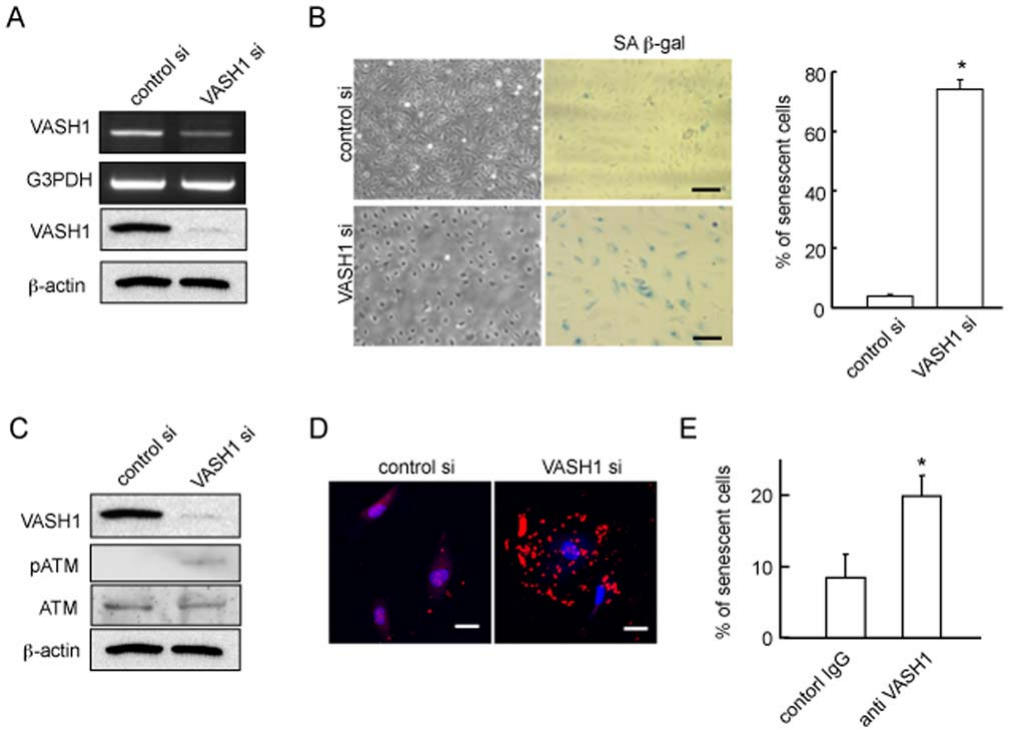
Knockdown of VASH1 induces premature senescence and enhances stress-induced cell death of HUVECs. (A) HUVECs were transfected with VASH1 siRNA or control siRNA. After a 24-hour incubation, RT-PCR and Western blotting for VASH1 were performed. (B) Phase-contrast photomicrographs (on the left: 48 hours after siRNA transfection) and SA beta-gal staining (on the right: 5 days after siRNA transfection) are shown. Scale bars are 250 microm. SA beta-gal-positive HUVECs were quantified, and the % senescent cells was calculated. Values are the ratio of SA beta-gal-positive cells to total cells, and are means and SDs of 3 wells. (*P<0.01, N = 3). (C) HUVECs were transfected with VASH1 or control siRNA. After a 24-hour incubation, Western blotting for VASH1, ATM and p-ATM was performed. (D) HUVECs were transfected with VASH1 siRNA or control siRNA. After a 24-hour incubation, LC3 (red) was immunostained. Scale bars are 25 microm. (E) HUVECs were cultured in growth medium with 100 microM H_2_O_2_ including mouse IgG (control) or 10 microg/ml VASH-1 antibody (4E12) for 48 h. Trypan blue exclusion assay was performed. Blue-stained cells quantified, and the % of dead cells was calculated (*P<0.01, N = 3). All the studies were repeated at least 3 times to confirm the reproducibility.

To understand the function of VASH1, we applied siRNA-mediated knock-down of VASH1 expression in HUVECs (Fig. 1A). We noticed that HUVECs lacking VASH1 became flatter under the basal condition (Fig. 1B). As this phenotype resembles that of senescent cells, we assessed the cellular senescence. There was a significant increase in SA β-gal reactivity in HUVECs lacking VASH1 (Fig. 1B). As ATM is known to be phosphorylated during premature senescence of ECs [14], we examined the level of phosphorylated ATM (p-ATM) in HUVECs lacking VASH1 and found an increase in it (Fig. 1C). It was described that autophagy is associated with premature senescence [15]. Immunostaining for LC3, a marker of autophagy, revealed autophagy in HUVECs lacking VASH1 (Fig. 1D). These results indicate that the knock-down of VASH1 caused the premature senescence of ECs. Although VASH1 lacks a classical signal sequence, it is secreted by binding to the small vasohibin-binding protein [16]. Accordingly, treatment of HUVECs with blocking monoclonal anti-VASH antibody induced a similar senescence phenotype (Fig. 1E), suggesting the importance of secreted VASH1 in protecting against cellular senescence.

**Figure 2:**
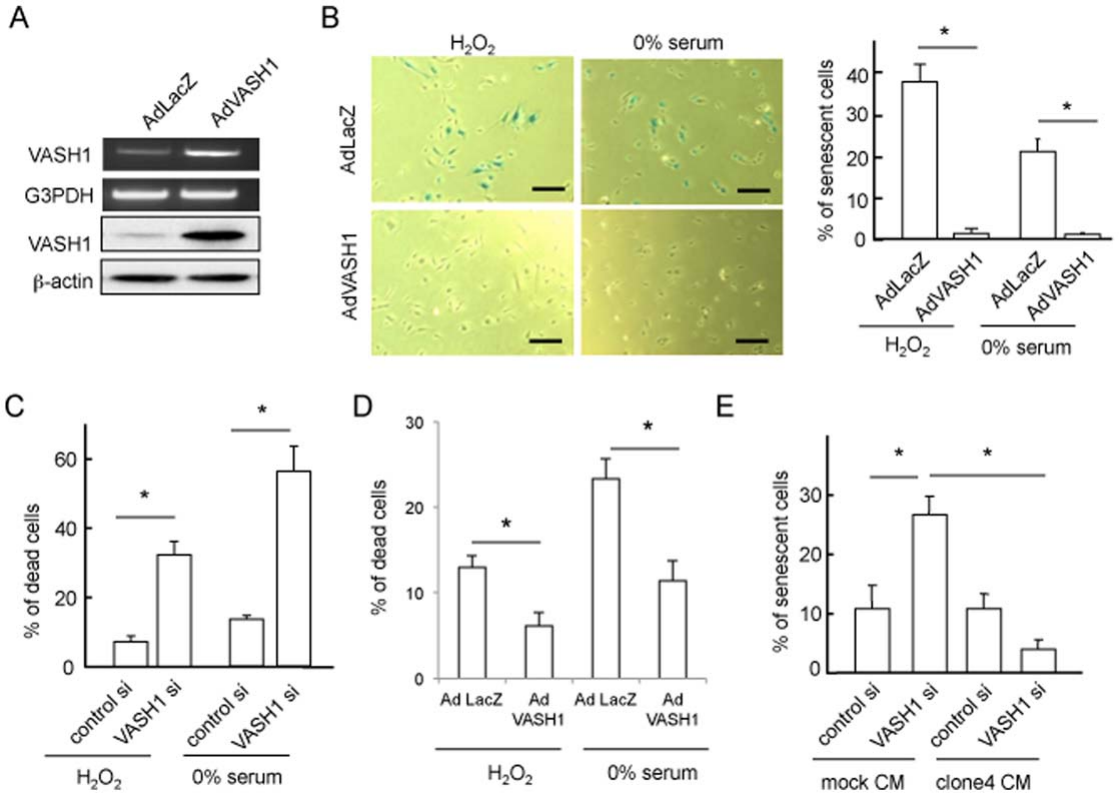
Overexpression of VASH1 inhibits premature senescence and cell death of HUVECs induced by cellular stresses. (A) HUVECs were infected with AdVASH1 or AdLacZ. After a 24-hour incubation, RT-PCR and Western blotting for VASH1 were performed. (B) HUVECs infected with AdVASH1 or AdLacZ were exposed to 100 µmol/L H_2_O_2_ for 1 hour, followed culture for 24 hours (on the left) or to 0% FCS/αMEM 24 hours (on the right). After a 6-day culture, SA β-gal staining was performed. Scale bars are 250 microm. SA β-gal-positive HUVECs were quantified, and the % of senescent cells was calculated (*P<0.01, N = 3). (C) HUVECs were transfected with VASH1 siRNA or control siRNA. After a 24-hour incubation, HUVECs were exposed to 100 µmol/L H_2_O_2_ or to 0% FCS/αMEM for 24 hours, and then the trypan blue exclusion assay was performed. Blue-stained cells were quantified, and the % of dead cells was calculated (*P<0.01, N = 3). (D) HUVECs infected with AdVASH1 or AdLacZ were exposed to 100 µmol/L H_2_O_2_ for 48 hours or to 0% FCS/αMEM for 24 hours for 24 hours; and then the trypan blue exclusion assay was performed (*P<0.01, N = 3). (E) HUVECs were transfected with VASH1 siRNA or control siRNA. After a 24-hour incubation, the growth medium was replaced with 50% conditioned medium derived from mock or VASH1 over-expressing MS1 clone 4. After a 24-hour incubation, the trypan blue exclusion assay was performed (*P<0.01, N = 3). All the studies were repeated at least 3 times to confirm the reproducibility.

We next overexpressed the human *VASH1* gene in HUVECs (Fig. 2A). Basal expression of VASH1 in ECs varies depending on the culture condition, as sparse HUVECs express less whereas subconflunet to confluent HUVECs express more VASH1 [3]. We therefore used sparse HUVECs for the overexpression. When those HUVECs were exposed to H_2_O_2_ or serum starvation, the AdVASH1-infected HUVECs exhibited resistance to premature senescence (Fig. 2B). We examined cell death after the exposure to cellular stresses. When HUVECs lacking VASH1 were exposed to H_2_O_2_ or serum starvation, they were vulnerable and easier to be killed by those stresses (Fig. 2C). In contrast, HUVECs overexpressing VASH1 were resistant to those cellular stresses (Fig. 2D). Importantly, stress-induced HUVEC premature senescence could be prevented by medium conditioned by stable VASH1 transfectant (Fig. 2E).

**Figure 3:**
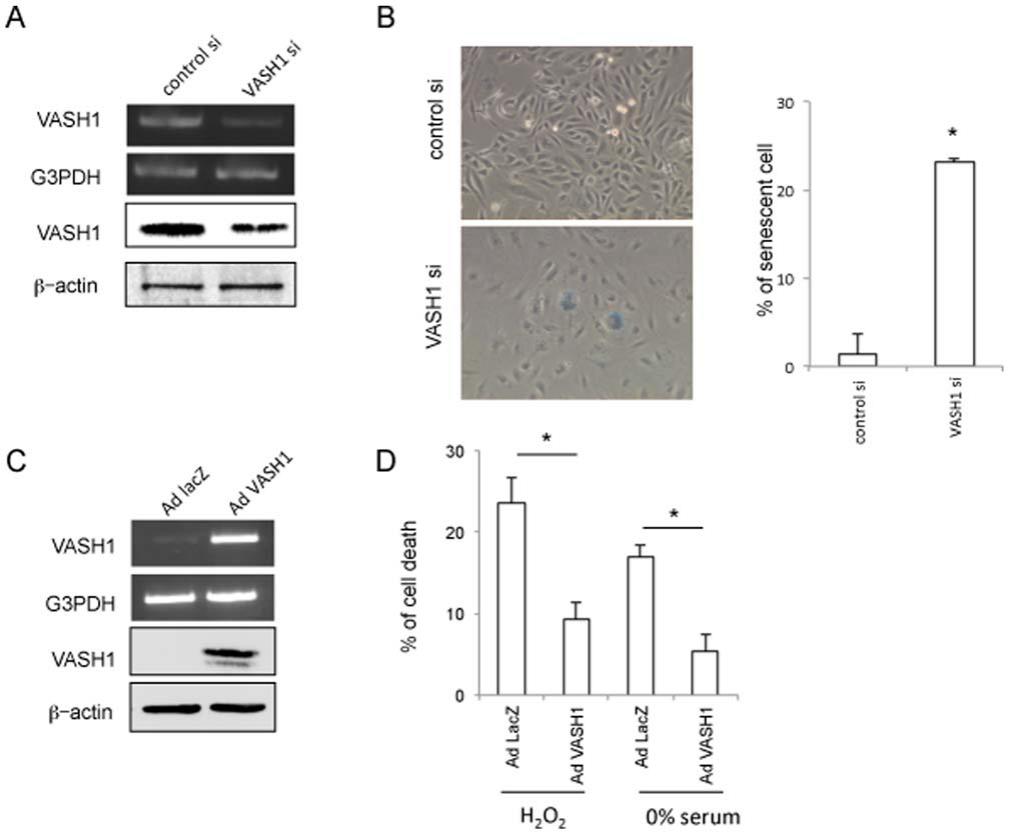
VASH1 inhibits premature senescence and cell death of HAECs. (A) HAECs were transfected with VASH1 siRNA or control siRNA. After a 24-hour incubation, RT-PCR and Western blotting for VASH1 were performed. (B) After a 6-day incubation, SA beta-gal staining was performed on HAECs that had been transfected with VASH1 siRNA or control siRNA. SA beta-gal-positive HAECs were quantified, and the % of senescent cells was calculated (*P<0.01, N = 3). (C) HAECs were infected with AdVASH1 or control AdLacZ. After a 24-hour incubation, RT-PCR and Western blotting for VASH1 were then performed. (D) HAECs infected with AdVASH1 or control AdLacZ were exposed to 100 µmol/L H_2_O_2_ or to 0% FCS/DMEM for 24 hours. The trypan blue exclusion assay was performed to judge cell death (*P<0.01, N = 3). All the studies were repeated at least 3 times to confirm the reproducibility.

We applied HAECs as another primary ECs. Identical to HUVECs, HAECs became senescent when VASH1 was knocked-down (Fig. 3A and B). Alternatively, HAECs became resistant to cellular stresses when VASH1 was overexpressed (Fig. 3C and D). We previously established stable human VASH1 transfectants of MS1 [8]. Those stable transfectants were also resistant to cellular stresses (Figure S1 A and B).

Collectively, the above data showed VASH1 to protect ECs from premature senescence and to make them resistant to stress-induced cell death.

### VASH1 protein level is determined via HuR-mediated posttranscriptional regulation

**Figure 4:**
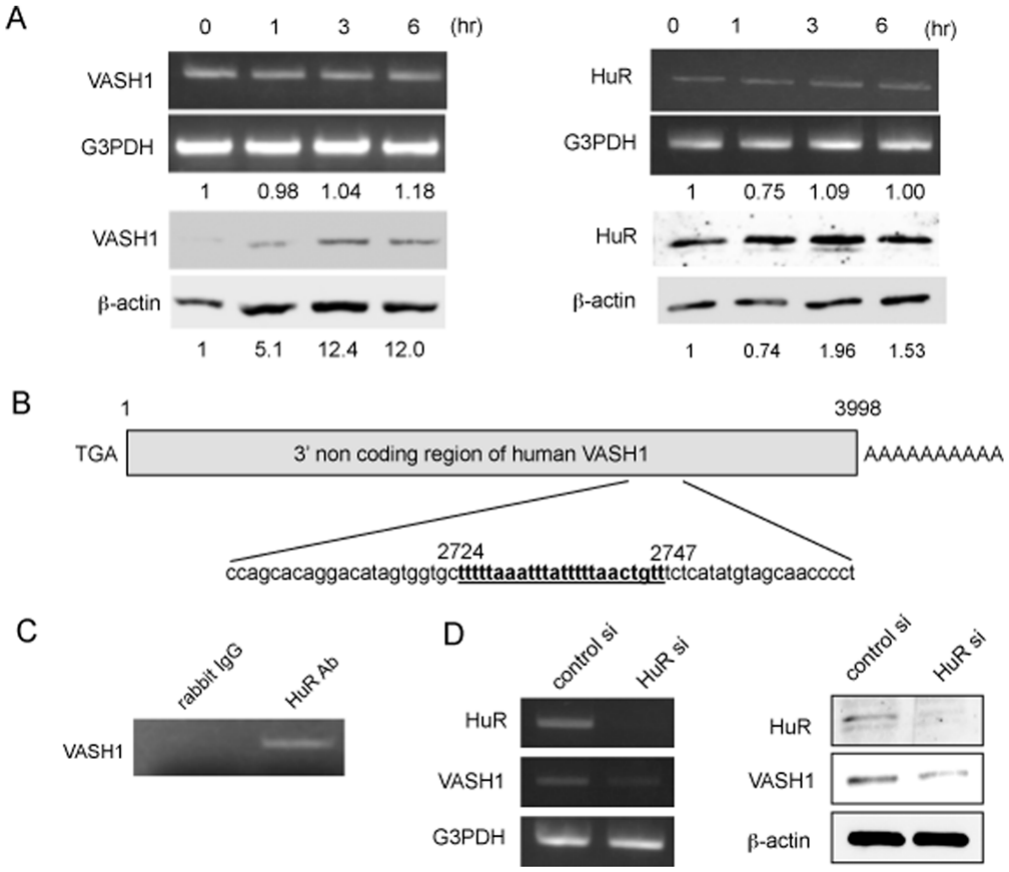
HuR increases VASH1 protein level in HUVECs. (A) HUVECs were incubated in 0% FCS/αMEM, and total RNA and protein were extracted at the indicated time points. Thereafter, RT-PCR and Western blotting for VASH1 were performed. Values below each band represent the mean fold change in RNA or protein expression level compared with the cognate zero time. (B) The AU-rich element (ARE) in the 3′ non coding region of the VASH1 gene is shown. (C) Immunoprecipitation and reverse transcription-polymerase chain reaction were performed as described in Materials and Methods. (D) HUVECs were transfected with HuR siRNA or control siRNA. After a 24- hour incubation, total RNA and protein were extracted; and then RT-PCR for HuR and Western blotting for VASH1 were performed. All the studies were repeated at least 3 times to confirm the reproducibility.

We examined whether cellular stress modulated the expression of VASH1 in ECs. We observed that cellular stress increased VASH1 protein level at 1–6 hour time points without the increase of VASH1 mRNA (Fig. 4A), indicating that post-transcriptional regulation might operate to increase the level of VASH1 protein when ECs were exposed to stress. Hu proteins are RNA-binding proteins that bind to AU-rich elements (AREs) in the 3′ untranslated region (UTR) of mRNAs [17]. We could show such elements in the 3′ UTR of VASH1 (Fig. 4B). Among Hu proteins, HuR is mostly related to cellular stress responses [18]. ChIP assay proved that HuR protein bound to this region in HUVECs (Fig. 4C). Furthermore, the knock-down of HuR abrogated the increase in VASH1 protein upon cellular stress in HUVECs (Fig. 4D). These results suggest that VASH1 in the ECs was targeted by HuR.

### VASH1 protects ECs via the induction of SOD2 and SIRT1

VEGF produced by ECs is reported to be a survival factor for ECs themselves [19]. SU5416, a VEGF receptor kinase inhibitor, did not affect the basal expression of VASH1 in HUVECs, but induced EC death (Figure S2 A and B). This EC death could be diminished by AdVASH1, but the senescence phenotype induced by VASH1 siRNA could not be reversed by exogenous VEGF (Figure S2B and C). These results suggest that the effect of VASH1 may not have involved the VEGF signaling.

**Figure 5:**
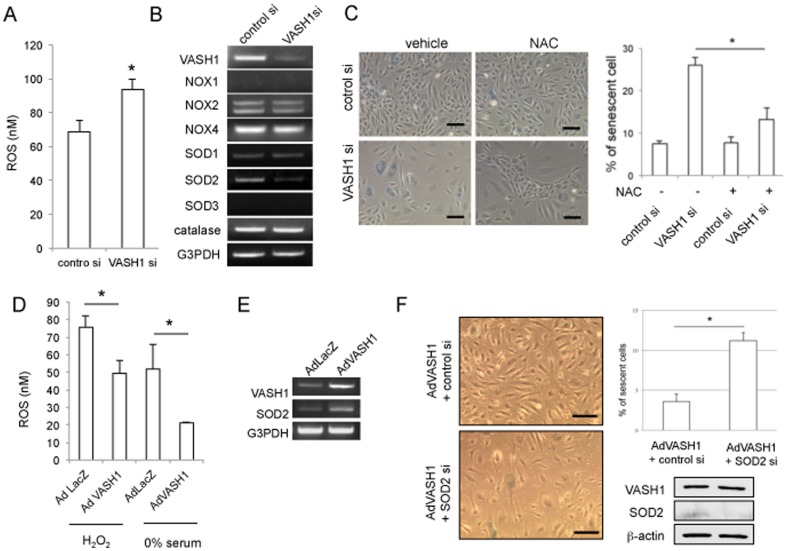
VASH1 controls SOD2 level and decreases ROS level in HUVECs. (A) HUVECs were transfected with VASH1 siRNA or control siRNA. After a 24-hour incubation, cellular ROS was determined as described in Materials and Methods (*P<0.01, N = 3). (B) HUVECs were transfected with VASH1 siRNA or control siRNA. Twenty-four hours later, RT-PCR for the indicated genes was performed. (C) HUVECs were transfected with VASH1 siRNA or control siRNA in the presence or absence of 50 µmol/L NAC. After a 12-hour incubation, the culture medium was replaced with growth medium containing vehicle or 50 µmol/L NAC. After a 6-day culture, SA β-gal staining was performed. Scale bars are 250 µm. SA β-gal-positive HUVECs were quantified, and the % of senescent cells was calculated (*P<0.01, N = 3). (D) HUVECs were infected with AdVASH1 or AdLacZ. After a 24-hour incubation, the cells were exposed to 100 µmol/L H_2_O_2_ for 1 hour or to 0% FCS/αMEM for 6 hours. Thereafter, the cellular ROS level was determined (*P<0.01, N = 3). (E) HUVECs were infected with AdVASH1 or AdLacZ. After a 24-hour incubation, RT-PCR for VASH1 and SOD2 was performed. (F) HUVECs were infected with AdVASH1 or AdLacZ. After a 24-hour incubation, HUVECs were then transfected with SOD2 siRNA or control siRNA. After a subsequent 24-hour incubation, the cells were exposed to 100 µ mol/L H_2_O_2_ for 1 hour followed by a 48-hour incubation in growth medium. Scale bars are 250 µm. SA β-gal staining and Western blotting for VASH1 and SOD2 were then performed. SA β-gal-positive HUVECs were quantified, and the % of senescent cells was calculated (*P<0.01, N = 3). All the studies were repeated at least 3 times to confirm the reproducibility.

One of the major causes of stress-induced premature senescence is ROS [20]. We could show that cellular ROS was significantly higher in HUVECs lacking VASH1 (Fig. 5A). We sought the reason for this increase in the ROS level in HUVECs lacking VASH1. Among various antioxidants tested, SOD2 was found to be down-regulated in HUVECs lacking VASH1 (Fig. 5B). Moreover, when ROS were quenched by NAC, premature senescence was partly but significantly inhibited in HUVECs lacking VASH1 (Fig. 5C). Alternatively, overexpression of VASH1 decreased the ROS level when exposed to cellular stresses, and up-regulated SOD2 in HUVECs (Fig. 5D and E). However, when this increase of SOD2 was knocked-down by siRNA, the protective effect of VASH1 on premature senescence was significantly abrogated (Fig. 5F).

**Figure 6:**
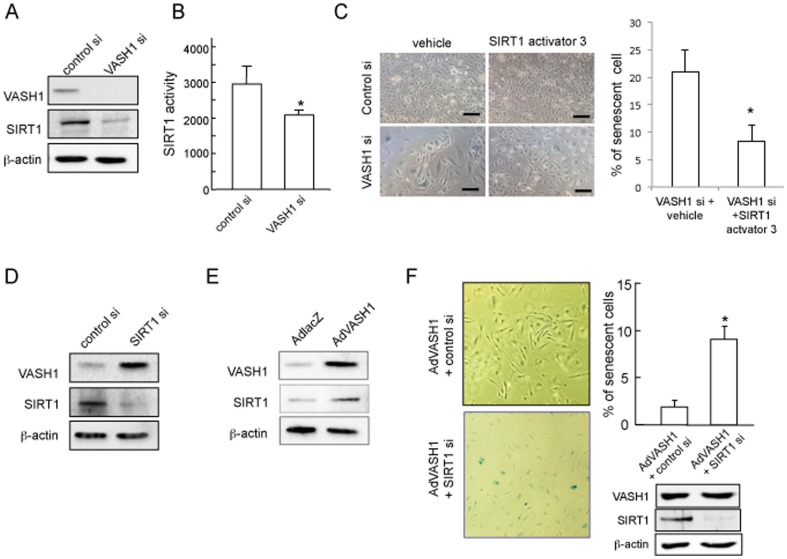
VASH1 controls SIRT1 level and increases stress resistance of HUVECs. (A) HUVECs were transfected with VASH1 siRNA or control siRNA. After a 72-hour incubation, Western blotting for VASH1 and SIRT1 was performed. (B) HUVECs were transfected with VASH1 siRNA or control siRNA. After a 24-hour incubation, SIRT1 activity was determined (*P<0.01, N = 3) (C) HUVECs were preteated with vehicle or 5 µmol/L SIRT1 activator 3 for 12 hours, and then transfected with VASH1 siRNA or control siRNA. After a 6-day incubation, SA β-gal staining was performed. Scale bars are 100 µm. SA β-gal-positive HUVECs were quantified, and the % of senescent cells was calculated (*P<0.01, N = 4). (D) HUVECs were transfected with SIRT1 siRNA or control siRNA. After a 72-hour incubation, Western blotting for VASH1 and SIRT1 was performed. (E) HUVECs were infected with AdVASH1 or AdLacZ. After a 72-hour incubation, Western blotting for VASH1 and SIRT1. (F) HUVECs were infected with AdVASH1. After a 24-hour incubation, HUVECs were then transfected with SIRT1 siRNA or control siRNA. After a subsequent 24-hour incubation, the cells were exposed to 100 µmol/L H_2_O_2_ for 1 hour followed by a 48-hour incubation in growth medium. Scale bars are 250 µm. SA β-gal staining and Western blotting for VASH1 and SIRT1 were then performed. β-gal-positive HUVECs were quantified, and the % of senescent cells was calculated (*P<0.01, N = 3). All the studies were repeated at least 3 times to confirm the reproducibility.

NAC partly inhibited premature senescence (Fig. 5B). Thus we reasoned that some additional mechanism might be involved. In addition to antioxidants, attention has been recently paid to the protective protein named SIRT1 [21]. Moreover, the synthesis and function of SIRT1 are related to HuR and ATM [22]. Therefore we tested the SIRT1 protein level. The knockdown of VASH1 significantly decreased the level of SIRT1 protein and its activity as well (Fig. 6A and B). However, if this reduced SIRT1 activity was enhanced by SIRT1 activator 3, the premature senescence of HUVECs lacking VASH1 was notably suppressed (Fig. 6C). Interestingly, the knock-down of SIRT1 increased the VASH1 protein level (Fig. 6D). Alternatively, when VASH1 was overexpressed in HUVECs by AdVASH1, SIRT1 protein significantly increased (Fig. 6E). Moreover, when SIRT1 was knocked-down, the VASH1-mediated protection against stress-induced premature senescence and cell death vanished (Fig. 6E and F).

Collectively, the above data showed VASH1 to protect ECs via the induction of SOD2 and SIRT1.

### VASH1 protects mice from death with acute lung injury induced by paraquat treatment

**Figure 7:**
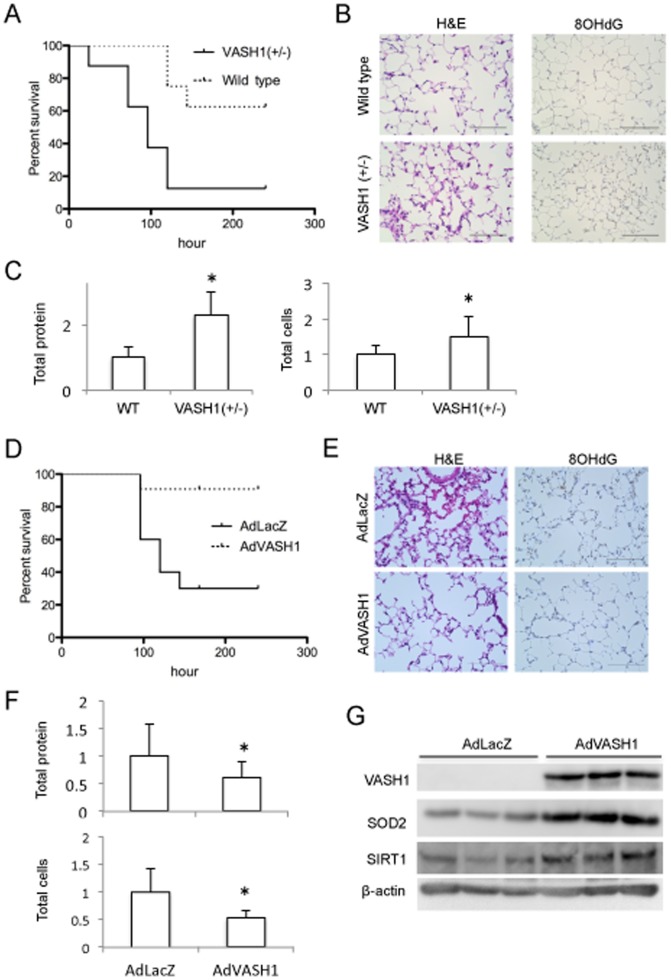
VASH1 protects mice from death with acute lung injury induced by paraquat treatment. (A) Paraquat was administered to WT (N = 8, dotted line) or *VASH1* (+/−) mice (N = 8, solid line), and the survival was observed over 10 days. Kaplan-Meier survival analysis showed significant difference. (B) Two days after paraquat administion, histological analyses of the lungs were performed. H&E staining is shown on the left, and immunostaining for 8-OHdG on the right. (C) Two days after paraquat administration, total protein and number of cells in the BALF were determined. *Significant difference (N = 7). (D) Paraquat was administered to *VASH1* (+/−) mice intratracheally infected with AdVASH1 (N = 10, dotted line) or AdLacZ (N = 11, solid line), and the survival was observed over 10 days. Kaplan-Meier survival analysis showed significant difference. (E) Two days after paraquat administion to AdLacZ or AdVASH1 mice, histological analyses of lungs were performed. H&E staining is shown on the left; and immunostaining fo 8-OHdG, on the right. (F) Two days after paraquat administration, BALF was collected; and total protein and number of cells in the BALF were determined. *Significant difference (N = 10). (G) Three days after the intratracheal infection of mice with AdVASH1 or AdLacZ, their lungs were removed and tissue extracts prepared. Western blotting for VASH1, SOD2, and SIRT1 in the extracts was performed.

To prove the protective role of VASH1 *in vivo*, we applied paraquat intoxication to mice. Paraquat is used as a redox cycler to stimulate superoxide production; and it causes acute organ injury mainly in the lungs [23]. Over a 10-day period following the administration of paraquat, the death rate even in the *VASH1* (+/−) KO mice was higher than that in the WT mice; and the survival curves for the WT and *VASH1* (+/−) KO mice were thus significantly different (Fig. 7A). Lungs were obtained 48 hours after the paraquat administration and histological analysis was performed. Cellular infiltration, and interstitial edema were evident in *VASH1* (+/−) KO mice (Fig. 7B, on the left). Immunohistochemistry for 8-OHdG revealed that DNA damage in *VASH1* (+/−) KO (Fig. 7B, on the right). Moreover, protein levels and cell counts in the bronchoalveolar lavage fluid (BALF) were significantly increased in *VASH1* (+/−) KO mice (Fig. 7C).

Staining for β-gal activity indicated that the intratracheally administered AdLacZ had been delivered to entire bronchial epithelium and some vessels (Figure S3 A and B). When AdVASH1 was intratracheally administered, VASH1 protein synthesis was detected in the lungs for at least 10 days (Figure S3C). We then tested whether the intratracheal administration of AdVASH1 could protect mice from the paraquat-induced lung injury. Over the same 10-day period, the deaths in the group of *VASH1 (+/−)* KO mice administered AdVASH1 were significantly fewer than in the group administered AdLacZ (Fig. 7D). Histological analysis of the lungs revealed that the AdVASH1 treatment attenuated the acute lung injury and DNA damage in *VASH1* (+/−) KO mice given AdVASH1 compared with that in those administered AdLacZ (Fig. 7E). Protein levels and cell counts in the BALF were significantly less in the former than in the latter (Fig. 7F). To verify the involvement of SOD2 and SIRT1 in the effect of VASH1 *in vivo*, we investigated those proteins in the lung tissue, and found that AdVASH1 increased SOD2 and SIRT1 contents in the lungs (Fig. 7G). Since the intratracheal administration of adenovirus vector tranfered gene mainly in bronchial epithelium, we assumed that VASH1 synthesized by bronchial epithelium affect on neighboring ECs. However, VASH1 might affect the bronchial epithelium as well. NHBECs did not express endogenous VASH1 (Figure S4A). In addition, the overexpression of VASH1 in NHBECs did not alter the level of SOD2 and SIRT1 (Figure S4B) nor the cell death after the treatment with H_2_O_2_ (Figure S4C). We therefore judge that the protective action of VASH1 in the paraquat-induced acute lung injury occurred mainly through ECs.

## Discussion

Our previous studies on VASH1 were focused on the inhibition of angiogenesis. Here we noticed, to our surprise, that the knock-down of basal VASH1 expression resulted in the premature senescence of ECs. We extended our study and revealed for the first time that VASH1 protected ECs from premature senescence and cell death when the cells were exposed to oxidative or serum-starvation stress. Angiogenesis inhibition generally causes vascular regression by inducing EC death, which regression may also result in proteinuria and hypertension *in vivo* [24]. We noted earlier that VASH1 neither instigates such vascular regression nor causes proteinuria and hypertension [25], [26]. Our present results further highlight the uniqueness of VASH1 in that it not only inhibited angiogenesis but also enhanced the maintenance of ECs by strengthening resistance against stress. The presice mechanism of the action of VASH1 remains to be elucidated.

VASH1 mRNA is induced by stimulation with angiogenic factors such as VEGF and FGF-2 via the activation of PKC-δ [27]. Here we observed that the VASH1 protein level increased without the increase in VASH1 mRNA when ECs were exposed to cellular stress, suggesting the posttranscriptional gene regulation. One of the important mechanisms of posttranscriptional regulation is the rapid degradation of mRNAs signaled by AREs in their 3′ UTR. The Hu family of RNA-binding proteins binds to AREs in the 3′UTRs of the target mRNAs, prevents their degradation, and enhances their translation [18]. There are 4 members of Hu proteins; HuB, HuC, HuD and HuR. Whereas HuB, HuC, and HuD are selectively expressed in the nervous system and play roles in neuronal differentiation and plasticity, HuR is ubiquitously expressed and exhibits numerous functions mostly related to cellular stress responses [18]. Thus, we consider the stress-induced VASH1 protein synthesis to have been regulated by HurR.

Here we gave evidence for 2 proteins as targets of VASH1 for the maintenance of ECs, the first being SOD2. The SOD family forms the major antioxidant defense system, which consists of 3 members: SOD1 as the cytoplasmic Cu/Zn-SOD, SOD2 as the mitochondrial Mn-SOD, and SOD3 as the extracellular Cu/Zn-SOD [28]. Because of its localization in mitochondria, SOD2 is the first line of defense against oxidative stress [29]. ECs are known to express a high level of SOD2 [30], and SOD2 is thought to play a principal role in protecting the vascular system from oxidative stress generated by various pathophysiological processes [31]. The second target of VASH1 we discovered was SIRT1. SIRT1 is a member of mammalian NAD^+^-dependent deacetylase family. Among them, SIRT1 is widely expressed, and is now considered to be responsible for the protection of cells from various types of stress [32]. Particularly, a number of reports indicate that vascular SIRT1 protects vessels from various vascular diseases including atherosclerosis and diabetic vascular complications [33]–[36]. The knock-down of VASH1 decreased the expression of SIRT1, whereas the knock-down of SIRT1 increased the expression of VASH1 in ECs. This may suggest that VASH1 lays upstream of SIRT1 in the axis of VASH1-SIRT1 in ECs. SIRT1 is expressed in ECs during angiogenesis [37]. The correlation of VASH1 and SIRT1 in the regulation of angiogenesis needs to be determined in future.

As mentioned earlier, angiogenesis inhibitors induce EC death and vascular regression [24]. Hence, the most intriguing aspect of VASH1 is simultaneous angiogenesis inhibition and EC protection. It is well documented that inflammatory cells form dense infiltrates at the site of angiogenesis and that oxidative stress is the major characteristic of such inflammatory conditions [38], [39]. Moreover, ROS can be one of the mediators of angiogenesis as well [27]. For that reason, we propose that the function of VASH1 is to halt angiogenesis and stabilize neo-vessels.

VASH1 is highly expressed in ECs at sites of angiogenesis. However, besides its presence there, we noticed previously that VASH1 protein is detectable in arterial ECs under the basal condition [40]. Arterial ECs are exposed to various physical forces. Moreover, oxidative stress-induced DNA damage is thought to play an important role in vascular senescence and senescence-related vascular diseases [41]. We therefore suggest that such VASH1 in the arterial wall is available there for the protection of vessels. Indeed we noted in earlier studies that VASH1 can prevent intimal thickening of arteries as well as diabetic renal injury [12], [42].

The lungs are the organ with the highest exposure to ambient air among all of the organs in the body. Because of its large alveolus surface and affluent blood perfusion, the lung tissue is most susceptible to oxidative injury. Here we used Paraquat to induce acute lung injury and showed that the intrabronchial sdministration of AdVASH1 protected lungs from acute lung injury. Since the intratracheal administration of adenovirus vector tranfered gene mainly in bronchial epithelium, we assumed that VASH1 synthesized by bronchial epithelium should affect on neighboring ECs in a paracrine manner. The excessive oxidative stress is thought to be one of the major causes of various lung diseases including chronic obstructive pulmonary diseases (COPD), pulmonary hypertension, and the post-reperfusion injury of transplanted lungs [43]–[45]. Moreover, there are several reports describing the relationship among SOD2, SIRT1, and COPD [46]–[48]. It would be therefore interesting to see if there is any relationship between those pulmonary diseases and VASH1.

In summary, our present study revealed that VASH1 not only inhibited angiogenesis but also enhanced the maintenance of ECs by strengthening their resistance against stress. We showed SOD2 and SIRT1 to be targets of VASH1 in ECs for strengthening this resistance. The close relationship among VASH1, SOD2 and SIRT1 may indicate the protective value of VASH1 in the vascular system.

## Supporting Information

Figure S1 **VASH1 over-expression inhibits the cell death of MS1 cells induced by cellular stresses.** Stable VASH1-expressing MS1 clones and mock MS1 transfectants were exposed to 200 µmol/L H_2_O_2_ (A) or serum starved (0% FCS/DMEM, B) for 24 hours. The trypan blue exclusion assay was then performed to judge cell death. Blue-stained cells were quantified, after which the % of dead cells was calculated. Values are the ratio of blue-stained cells to total cells, and are the means and SDs of 3 wells. *Significant difference compared with the value for the corresponding mock.
(TIFF)

Figure S2 **VASH1 protects HUVEC death by the treatment with a VEGF receptor inhibitor.** (A) HUVECs were incubated in growth medium containing SU5416. Total RNA and protein were extracted from the cells at the indicated times, and then RT-PCR and Western blotting for VASH1 were performed. (B) HUVECs were infected with AdVASH1 or AdLacZ. After a 24-hour incubation, SU5416 was added. After an additional 3 days' incubation, the trypan blue exclusion assay was performed. Blue-stained cells were quantified, and the % of dead cells was calculated. *Significant difference compared with the value for the corresponding AdLacZ. (C) HUVECs were transfected with VASH1 siRNA or control siRNA. In some case, VEGF (1 nmol/L) was added to HUVECs that had been transfected with VASH1 siRNA. The cells were cultured for 3 days, and observed by phase-contrast microscopy.
(TIFF)

Figure S3 **Intratracheal administration of adenovirus vector.** (A) PBS (left photo) or AdVASH1 (right photo) was intratracheally administered to WT mice. Twenty-four hours after the administration, the lungs were removed and processed for β-gal staining. (B) Microscopic observation showed that SA β-gal-positive cells were found mainly in the bronchial epithelium, but also in some blood vessels and interstitial macrophages. (C) After the intratracheal administration of AdVASH1 to WT mice, the lungs were removed at the indicated time points; and tissue extracts prepared from them were then Western blotted for VASH1.
(TIFF)

Figure S4 **VASH1 does not increase stress resistance of NHBECs.** (A) Expression of endogenous VASH1 in HUVECs and NHBECs was analyzed by RT-PCR. (B) NHBECs were infected with AdVASH1 or AdLacZ. After a 72-hour incubation, Western blotting for VASH1, SOD2, and SIRT1 was performed. (C) NHBECs were infected with AdVASH1 or AdLacZ. After a 72-hour incubation, NHBECs were exposed to 400 µmol/L H_2_O_2_ for 24 hours; and the trypan blue exclusion assay was then performed. Blue-stained cells were quantified, and the % of dead cells was calculated. Values are the ratio of blue-stained cells to total cells, and are the means and SDs of 4 wells.
(TIFF)
