# Supplementary figures and images for: Angiogenesis Inhibitor Vasohibin-1 Enhances Stress Resistance of Endothelial Cells via Induction of SOD2 and SIRT1
Source: PLoS One. 2012 Oct 8;7(10):e46459. doi: 10.1371/journal.pone.0046459 (PMC3466306; doi:10.1371/journal.pone.0046459)

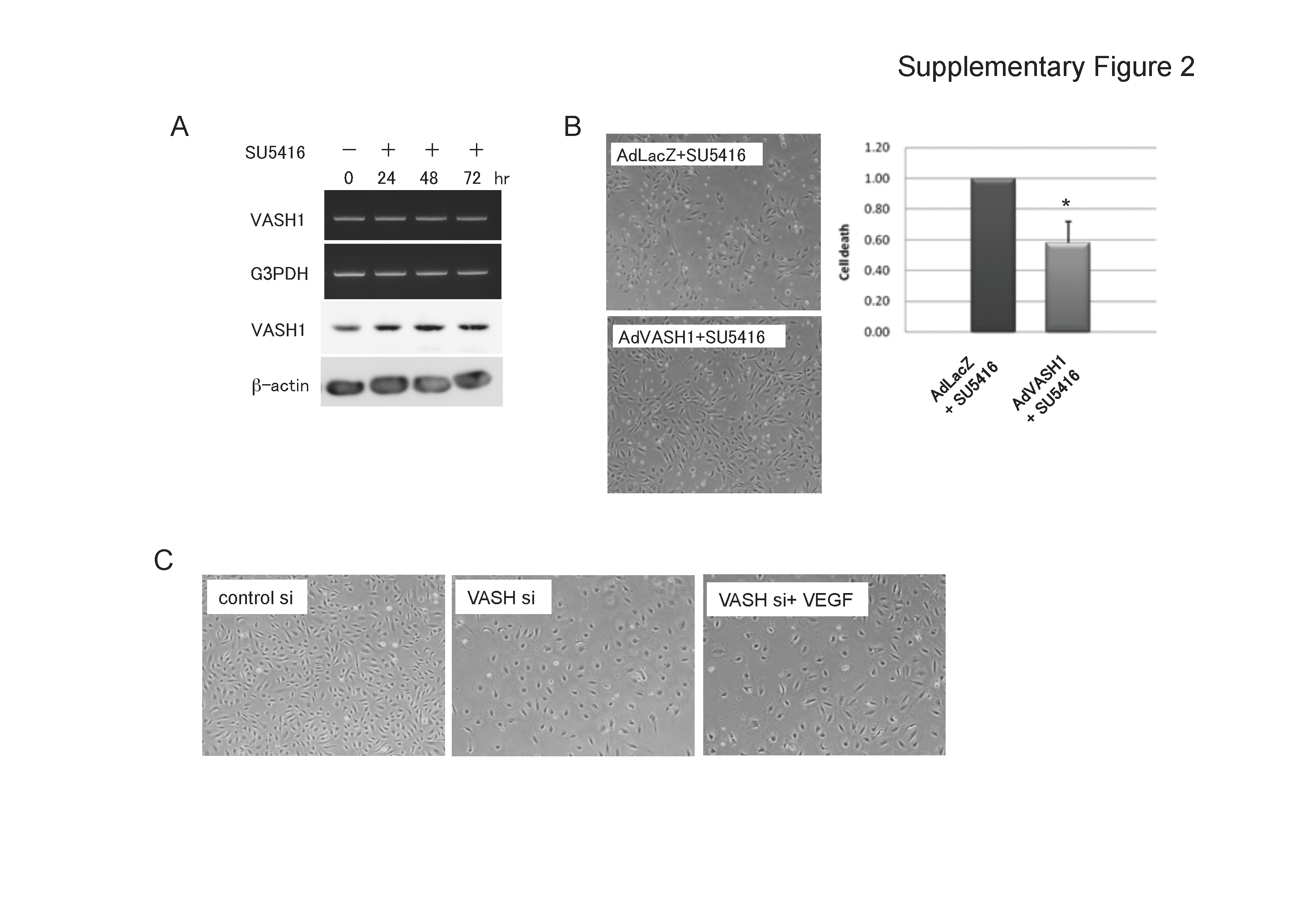

Supplement: Figure S2 — VASH1 protects HUVEC death by the treatment with a VEGF receptor inhibitor. (A) HUVECs were incubated in growth medium containing SU5416. Total RNA and protein were extracted from the cells at the indicated times, and then RT-PCR and Western blotting for VASH1 were performed. (B) HUVECs were infected with AdVASH1 or AdLacZ. After a 24-hour incubation, SU5416 was added. After an additional 3 days' incubation, the trypan blue exclusion assay was performed. Blue-stained cells were quantified, and the % of dead cells was calculated. *Significant difference compared with the value for the corresponding AdLacZ. (C) HUVECs were transfected with VASH1 siRNA or control siRNA. In some case, VEGF (1 nmol/L) was added to HUVECs that had been transfected with VASH1 siRNA. The cells were cultured for 3 days, and observed by phase-contrast microscopy. (TIFF) [file pone.0046459.s002.tiff]

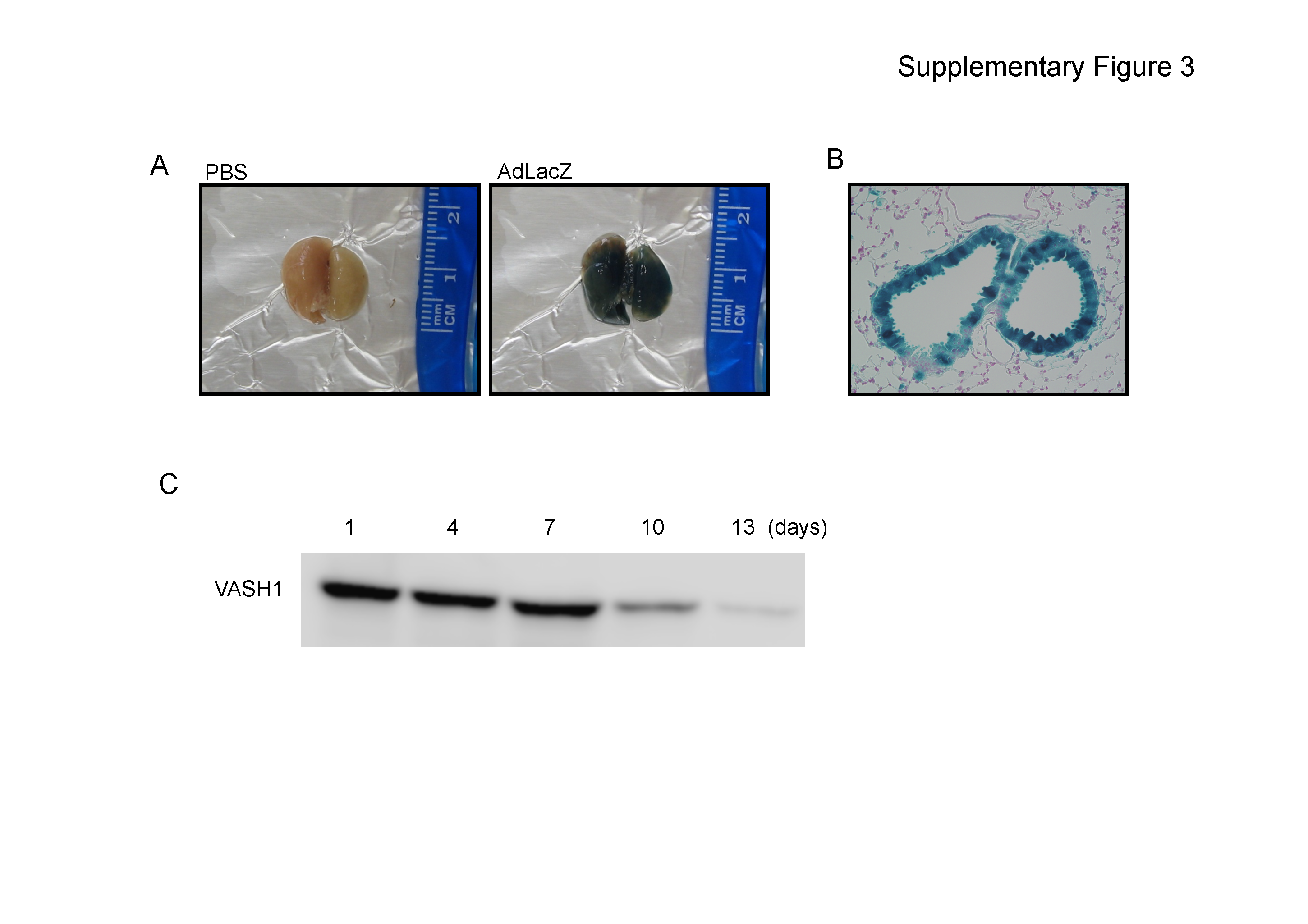

Supplement: Figure S3 — Intratracheal administration of adenovirus vector. (A) PBS (left photo) or AdVASH1 (right photo) was intratracheally administered to WT mice. Twenty-four hours after the administration, the lungs were removed and processed for β-gal staining. (B) Microscopic observation showed that SA β-gal-positive cells were found mainly in the bronchial epithelium, but also in some blood vessels and interstitial macrophages. (C) After the intratracheal administration of AdVASH1 to WT mice, the lungs were removed at the indicated time points; and tissue extracts prepared from them were then Western blotted for VASH1. (TIFF) [file pone.0046459.s003.tiff]

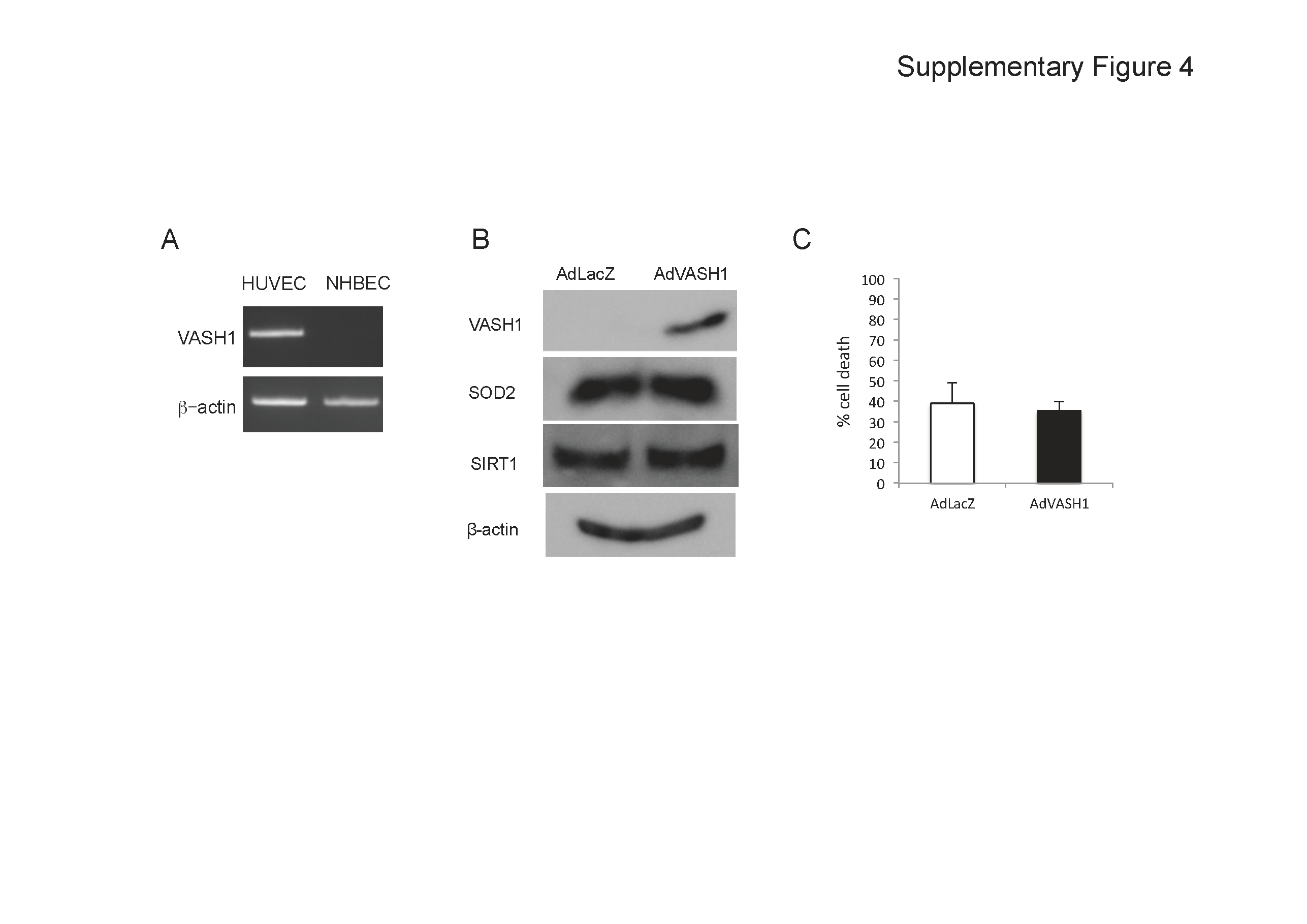

Supplement: Figure S4 — VASH1 does not increase stress resistance of NHBECs. (A) Expression of endogenous VASH1 in HUVECs and NHBECs was analyzed by RT-PCR. (B) NHBECs were infected with AdVASH1 or AdLacZ. After a 72-hour incubation, Western blotting for VASH1, SOD2, and SIRT1 was performed. (C) NHBECs were infected with AdVASH1 or AdLacZ. After a 72-hour incubation, NHBECs were exposed to 400 µmol/L H2O2 for 24 hours; and the trypan blue exclusion assay was then performed. Blue-stained cells were quantified, and the % of dead cells was calculated. Values are the ratio of blue-stained cells to total cells, and are the means and SDs of 4 wells. (TIFF) [file pone.0046459.s004.tiff]
